# Foot self-care competence reported by patients with rheumatoid arthritis: a cross-sectional study

**DOI:** 10.1186/s13047-022-00599-4

**Published:** 2022-12-17

**Authors:** Anne-Marie Laitinen, Miko Pasanen, Elina Wasenius, Minna Stolt

**Affiliations:** 1 Department of Nursing Science, University of Turku, Turku, 20014 Finland; 2 Turku University Hospital, Turku, Finland; 3 Podiatry, Metropolia University of Applied Sciences, Helsinki, Finland; 4 Department of Nursing Science, University of Eastern Finland, Kuopio, Finland

**Keywords:** Competence, Foot care, Foot self-care, Rheumatoid arthritis, Questionnaire

## Abstract

**Background:**

Foot self-care is important for preventing foot problems and maintaining one’s foot health. Foot self-care requires competence to identify foot problems, knowledge and skills to care for those problems, and a willing attitude to care for one’s foot health. However, there is major gap in the research evidence of foot self-care competence among patients with rheumatoid arthritis. This study aimed to analyse self-reported levels of competence in foot self-care among patients with rheumatoid arthritis.

**Methods:**

A cross-sectional study design was used. Data were collected using a survey consisting of a self-reported competence in foot self-care competence scale (response options on 5-point Likert scale, higher values indicate higher competence) and background questions. The data were analysed with descriptive and inferential statistics and the psychometric properties of the scale using Rasch analysis.

**Results:**

The participants’ (*n* = 251) self-reported level of competence in foot self-care was moderate (mean 3.50, standard deviation [SD], 0.66). On the sum variable level, the highest mean score was for attitude towards foot self-care (3.98; SD, 0.69), followed by foot self-care knowledge (3.45; SD, 0.67) and experience providing foot self-care (3.38; SD, 0.69). Higher self-reported foot self-care knowledge and female sex were associated with higher self-reported competence in every sum variable.

**Conclusions:**

Patients with rheumatoid arthritis evaluated their level of competence in foot self-care as moderate and some deficiencies were identified. These results indicate the importance of educating patients with rheumatoid arthritis about how to advance their foot self-care skills and knowledge. In the future, patients with rheumatoid arthritis could benefit from interventions that increase their knowledge of foot self-care together with practical examples, such as online videos, that demonstrate the practical conduct of foot self-care skills in daily life.

## Background

Foot self-care is important for preventing foot problems and maintaining one’s foot health. Foot self-care requires competence to identify foot problems, knowledge and skills to care for those problems, and a willing attitude to care for one’s foot health. Living with long-term health problems increases the importance of foot self-care [1, 2]. Rheumatoid arthritis (RA) is a long-term disease that in some cases cause significant foot problems [3]. However, little is known about self-assessed foot self-care competence among patients with RA (PwRA).

RA, an inflammatory autoimmune disease that causes joint stiffness, dysfunction, and dislocation, often appearing first in the joints of the feet [4]. The prevalence of RA increases with age and its global prevalence is approximately 0.25% [5, 6]. The vast majority of PwRA report foot health problems [7]. Foot joint stiffness and swelling are common at the onset of RA [8]. As RA progresses, foot problems tend to increase [9] and lower-extremity function gradually declines [10]. The most common foot problems are joint disorders, toenail problems such as thickened toenails and skin problems such as corns and calluses on the toes and soles of the feet [7, 11]. Moreover, foot pain in PwRA is reported to be prevalent and in some studies even 70% of PwRA is reporting persistent foot pain [3, 12].

Active and regular foot self-care is important for maintaining one’s foot health and preventing the deterioration of existing foot problems [13]. Despite the importance of foot self-care, PwRA appreciate their foot health but tend to consider their foot self-care ability decreasing as RA progresses [14]. PwRA are motivated to care for their feet; however, they are unsure of their skills and knowledge of how to properly provide such care [15]. In addition to skills and knowledge, some physical factors, such as obesity [16], poor eyesight and decreased manual dexterity [17] may affect foot self-care. Foot self-care in this study refers to activities that an individual performs on his or her feet, including daily foot hygiene, skin and nail care, use of suitable and correct sized footwear and socks and lower-limb exercises [18]. Competence in this study was defined as knowledge, skills, values, attitudes and experiences [19, 20] in the context of foot self-care. Foot self-care competence in this study means, that a person has knowledge for foot self-care, skills to identify and care for foot problems, a willing attitude towards foot self-care and experience of foot self-care.

Previous research generally focused on the concept of foot self-care rather than on providing evidence of competence. This research was predominantly conducted among patients with diabetes mellitus (DM), who have knowledge how to self-care for their feet; however, their skills or activities are often inconsistent or outdated [21, 22] and the frequency of foot self-care activities are unsystematic [22, 23]. Particularly among patients with DM, female sex [24] and higher foot care knowledge levels [25] are associated with self-care competence. Moreover, patients with a longer history of living with DM assessed their feet regularly and cared more actively for them than patients with newly diagnosed DM [23]. Similar to DM, RA is a long-term health condition [4]. However, there is major gap in the research evidence of foot self-care competence among PwRA. To promote foot health among the patients, identifying areas for patient education and foot self-care interventions and reframing existing foot health services based on evidence of foot self-care competence from PwRA are needed.

This study aimed to analyse self-reported levels of competence in foot self-care among patients with RA to provide evidence for the development of foot health services in this population.

The research questions were:

1. What are the self-reported foot self-care competence levels among PwRA?
2. What factors, if any, are associated with self-reported foot self-care competence levels among PwRA?

## Methods

A cross-sectional study design was applied.

### Data collection

Data were collected from a regional patient organisation in Finland of approximately 1500 members who are diagnosed with rheumatic diseases. The organisation aims to provide support and information about living with long-term diseases and provide social and rehabilitative activities for its members. A paper questionnaire accompanied by a prepaid return envelope was sent to all adult members (*N* = 1318) of the association. The data were collected in January and February of 2019. A total of 504 responses were obtained, of which 251 were from PwRA.

### Instrument

The Competence in Foot Self-care Scale instrument was developed for the purposes of this study to measure self-evaluated foot self-care competence levels. First, a systematic literature search was conducted to identify any existing foot self-care competence instruments. None of the identified instruments measured foot self-care competence; instead, they focused only on self-care knowledge or the frequency that foot self-checks or self-care was performed. To focus on competence from a larger perspective, the theoretical structure of the instrument followed the basic competence definition by Lakanmaa and colleagues [20]: knowledge, skills, attitudes and values and experience. Accordingly, items related to the identification and care of the skin, nail, foot structure and pain were generated based on previous research and evaluated by the research team. A preliminary version of the instrument was pilot-tested with a sample of PwRA (*n* = 20). No modifications were made to the method. The final instrument consisted of 32 items measuring the identification and care of the skin, nail and foot structural problems and foot pain. The items were divided into four categories: knowledge (eight items), skills (eight items), attitude (eight items) and experience (eight items). Each category is further divided into identification and care. Responses were rated using a 5-point Likert scale (from 1 = very poorly to 5 = very well).

Several background questions were posed. Data on age, sex, education and employment status were collected as sociodemographic information. Background questions focusing on foot health included participants’ evaluations of the importance of their foot health in general, consultation with medical or health care professionals due to foot problems, self-evaluated foot self-care knowledge level and sufficiency of foot self-care patient education.

### Data analysis

The data were analysed statistically using IBM SPSS Statistics 26.0 for Windows (IBM Corp, Armonk, NY, USA) and Winsteps 4.8.1.0. Descriptive statistics (frequency, percentage, mean and standard deviation) were calculated. Second, the sum of the variables was formed based on the theoretical dimensions of the instrument. Third, the associations between foot self-care competence and its sum variables with background variables were analysed using Spearman’s correlation coefficient and analysis of variance. Associated background factors (sex, education, importance of foot health, self-evaluated foot self-care knowledge level) to foot self-care competence sum variables were included in the multivariate linear regression model. A model used each foot self-care competence sum variable as a dependent variable and included the associated background factors. Statistical significance was set at p < 0.05. Finally, the psychometric properties of the Competence in Foot Self-care Scale were examined with Rasch analysis, focusing on category functioning, unidimensionality, item fit, and person and item separation [26].

### Ethical considerations

Ethical approval was obtained from the university ethical review board (8/2018, 29.1.2018). The study followed good scientific practices in all phases [27]. Each eligible participant received an information letter stating the study purpose, data collection procedures, anonymity and participation confidentiality. Each participant gave informed written consent to participate prior to entering the study.

## Results

### Participants’ background characteristics

The median participant age (*n* = 251) was 69 years (range, 21–86) and the majority of them were female (*n* = 216 [87%]; Table 1). Most participants were retired. Overall, the PwRA considered foot health very important (73%). Half of the participants (56%) sought medical or health care for their foot problems. The participants considered their foot self-care knowledge as good (49%) and slightly over half (54%) reported receiving sufficient foot self-care patient education from health care professionals.

**Table 1:** Participants’ characteristics (*n* = 251)

Background variables	f	%
Age, years	Median, 69 (interquartile range, 14)	
Sex		
Female	216	87
Male	33	13
Education		
Four to eight years of elementary school	85	34
Nine years of elementary school	90	36
High school	76	30
Employment status		
Manager	7	3
Employee	61	24
Entrepreneur	8	3
Retired	172	69
Unemployed	2	1
Self-perceived importance of foot health		
Very important	184	73
Important	54	22
Somewhat important	13	5
Sought professional health care for foot problems		
Yes	139	56
No	111	44
Self-evaluated foot self-care knowledge level		
Very good	16	6
Good	123	49
Not good, not poor	94	38
Poor	16	6
Very poor	1	1
Received sufficient foot self-care patient education from health care professional		
Yes	112	46
No	133	54

### Self-reported foot self-care competence level

The overall self-reported foot self-care competence level was moderate (mean 3.50, SD 0.66; Table 2). On the sum variable level, the highest mean was noted for attitude towards foot self-care (3.98; SD, 0.69), followed by knowledge of foot self-care (3.45; SD, 0.67) and experience performing foot self-care (3.38; SD, 0.69). The weakest mean score was noted for foot self-care skills (3.35; SD, 0.65).

**Table 2:** Participants’ (*n* = 251) self-reported foot self-care competence

Abbreviated item		**1**	**2**	**3**	**4**	**5**	**Mean**	**SD**
	***n***	**f (%)**	**f (%)**	**f (%)**	**f (%)**	**f (%)**		
**COMPETENCE (total)**	*244*						3.50	0.66
**FOOT SELF-CARE KNOWLEDGE**	*248*						3.45	0.67
**I know how to identify:**								
Changes in the foot skin	*245*	2 (1)	18 (7)	63 (25)	137 (55)	25 (10)	3.67	0.79
Changes in the toenails	*242*	2 (1)	20 (8)	57 (23)	137 (55)	26 (10)	3.68	0.80
Structural changes in the feet and toes	*244*	6 (2)	31 (12)	81 (32)	107 (43)	19 (8)	3.42	0.90
Need to care for foot pain	*245*	2 (1)	29 (12)	70 (28)	116 (46)	28 (11)	3.58	0.87
**I know how to care for:**								
Changes in the foot skin	*243*	3 (1)	27 (11)	84 (34)	114 (45)	15 (6)	3.47	0.83
Changes in the toenails	*244*	4 (2)	38 (15)	90 (36)	100 (40)	12 (5)	3.32	0.86
Structural changes in the feet and toes	*243*	6 (2)	53 (21)	99 (39)	73 (29)	12 (5)	3.14	0.91
Foot pain	*246*	5 (2)	38 (15)	92 (27)	91 (36)	20 (8)	3.36	0.91
**FOOT SELF-CARE SKILLS**	*246*						3.35	0.65
**I can identify:**								
Changes in the foot skin	*244*	2 (1)	16 (6)	57 (23)	151 (60)	18 (7)	3.68	0.75
Changes in the toenails	*243*	1 (1)	17 (7)	62 (25)	142 (57)	21 (8)	3.67	0.75
Structural changes in the feet and toes	*242*	4 (2)	30 (12)	78 (31)	116 (46)	14 (6)	3.41	0.85
Need to care for foot pain	*245*	1 (1)	24 (10)	72 (29)	120 (48)	28 (11)	3.62	0.82
**I can care for:**								
Changes in the foot skin	*242*	6 (2)	42 (17)	87 (35)	95 (38)	12 (5)	3.27	0.89
Changes in the toenails	*241*	8 (3)	54 (22)	95 (38)	76 (30)	8 (3)	3.09	0.91
Structural changes in the feet and toes	*242*	21 (8)	66 (26)	90 (34)	58 (23)	7 (3)	2.86	0.99
Foot pain	*244*	15 (6)	45 (18)	93 (37)	74 (26)	17 (7)	3.15	1.00
**ATTITUDE TOWARDS FOOT SELF-CARE**	*244*						3.98	0.69
**I think it is important that I can identify:**								
Changes in the foot skin	*238*	1 (1)	8 (3)	31 (12)	136 (54)	62 (25)	4.06	0.73
Changes in the toenails	*240*	2 (1)	5 (2)	36 (14)	139 (55)	58 (23)	4.04	0.70
Structural changes in the feet and toes	*241*	1 (1)	11 (4)	40 (169)	131 (52)	58 (23)	3.99	0.76
Need to care for foot pain	*240*	1 (1)	6 (2)	40 (16)	125 (50)	68 (27)	4.08	0.74
**I think it is important that I can care for:**								
Changes in the skin of the foot	*240*	2 (1)	6 (2)	39 (16)	139 (55)	54 (22)	3.99	0.73
Changes in the toenails	*239*	2 (1)	6 (2)	43 (17)	135 (54)	53 (21)	3.97	0.74
Structural changes in the feet and toes	*238*	2 (1)	13 (5)	51 (20)	120 (48)	52 (21)	3.87	0.83
Foot pain	*243*	3 (1)	6 (2)	47 (19)	126 (50)	61 (24)	3.98	0.78
**FOOT SELF-CARE EXPERIENCE**	*244*						3.38	0.85
**I have experience identifying:**								
Changes in the skin of the foot	*241*	10 (4)	35 (14)	66 (27)	99 (40)	31 (12)	3.45	1.02
Changes in the toenails	*241*	10 (4)	31 (12)	76 (30)	94 (38)	30 (12)	3.43	1.01
Structural changes in the feet and toes	*241*	12 (5)	35 (14)	81 (32)	84 (34)	29 (12)	3.35	1.04
Need to care for foot pain	*243*	7 (3)	27 (11)	69 (28)	97 (39)	43 (17)	3.60	1.00
**I have experience caring for:**								
Changes in the skin of the foot	*240*	15 (6)	31 (12)	70 (28)	95 (38)	29 (12)	3.40	1.07
Changes in the toenails	*241*	12 (5)	40 (16)	80 (32)	85 (34)	24 (7)	3.28	1.03
Structural changes in the feet and toes	*240*	18 (7)	44 (18)	89 (36)	68 (27)	21 (8)	3.12	1.06
Foot pain	*243*	12 (5)	34 (14)	76 (30)	85 (34)	36 (14)	3.42	1.07

Response scale: 1 = very poor, 2 = poor, 3 = not poor, not well, 4 = well, 5 = very well

At the item level, the highest mean scores were for importance in identifying foot pain (4.08; SD, 0.74). Instead, related to self-reported competence in caring for foot problems, the participants reported low values for self-care structural deformities of the feet and toes (2.86; SD, 0.99).

### Factors associated with foot self-care competence level

Background factors associated with foot self-care competence included higher self-reported foot self-care knowledge and female sex for every sum variable (Table 3). Higher values of perceived importance of foot health were related to higher competence in knowledge, attitude and experience. Receiving sufficient self-care education from health care professionals was associated with higher competence in knowledge, skills and experience. Age was related to higher self-reported competence in attitude (*p* < 0.001) and experience (*p* = 0.048). Basic education was associated with higher attitude values (*p* < 0.001) for all sum variables.

**Table 3:** Influence of background factors on foot self-care competence sum variables determined by a multivariate linear regression model

	**Foot self-care competence**											
	**Knowledge**			**Skills**			**Attitude**			**Experience**		
	**ß**	**SE**	***p***	**ß**	**SE**	***p***	**ß**	**SE**	***p***	**ß**	**SE**	***p***
Intercept	34.308	2.313	< 0.001	33.192	2.248	< 0.001	33.912	2.279	< 0.001	34.243	3.126	< 0.001
Sex^a^	1.244	0.933	0.184	0.975	0.907	0.283	0.575	0.918	0.532	1.656	3.126	0.191
Education												
9 years of elementary^a^	-0.030	0.731	0.967	0.342	0.711	0.631	3.386	0.720	< 0.001	-0.503	0.989	0.611
High school	-0.955	0.750	0.204	0.830	0.729	0.256	3.380	0.739	< 0.001	-0.308	1.013	0.762
Importance of foot health	-0.685	0.556	0.219	0.176	0.541	0.746	-1.820	0.548	< 0.001	0.981	0.752	0.193
Self-evaluated foot self-care knowledge	-3.149	0.447	< 0.001	-3.248	0.434	< 0.001	-1.170	0.441	< 0.001	-3.574	0.604	< 0.001

^a^The reference category for sex is male, while that for education is the 4–8 years of elementary school

Based on the generated linear regression models, the lower the participants’ self-evaluated foot self-care knowledge, the poorer their values for the foot self-care knowledge sum variable (β = -3.149; *p *< 0.001), foot self-care skills sum variable (β = -3.248; *p* < 0,001), attitude towards foot self-care sum variable (β = -1.17; *p* < 0.001) and foot self-care experience sum variable (β = -3.574; *p* < 0.001). Regarding the attitude towards foot self-care sum variable, the values were higher among those participants who completed 9 years of elementary school education (β = 3.386; *p* < 0.001) or a high school education (β = 3.380; *p *< 0.001) compared to those who completed 4–8 years of elementary school education. In addition, for the attitude towards foot self-care sum variable, the values were lower when participants considered their foot health unimportant (β = -1.820; *p *< 0.001) or had low self-evaluated foot self-care knowledge (β = -1.170; *p* < 0.001).

## Discussion

PwRA self-reported moderate foot self-care competence levels. Previous studies reported evidence of foot self-care knowledge and skills; however, comprehensive analyses of foot self-care competence are lacking. This study fills this gap by producing new findings on self-reported foot self-care competence from four areas: knowledge, skills, attitudes and experience. Our results are unique to PwRA. PwRA evaluated their foot self-care knowledge levels and attitudes as being higher than their skill levels. These results are in line with those of studies focusing on patients with DM [21, 22].

The reason for the low evaluation of self-care skills could be deficiencies in foot health-related patient education. PwRA have complained of inequality accessing podiatry services [14], that health care professionals do not always pay attention to foot health-related patient education and that practical guidance or demonstrations on how to correctly care for one’s feet are lacking [21, 28, 29]. In the future, it would be beneficial if care guidelines or recommendations would include specific information on how health care professionals should perform foot health assessments and when referring the patient to the podiatrist. These care guidelines could even include modern sources of information, such as evidence-based websites that include short video clips, to direct the patient towards proper foot self-care. In addition to deficits in foot self-care knowledge, PwRA could have physical restrictions that prevent them from caring for their own feet [17] or limited motivation about foot self-care [14]. To encourage patients to provide foot self-care and promote their competence, PwRA were offered participation in guidance groups held by a podiatrist in which they would receive empowering practical education and information regarding foot self-care. These guidance groups would be cost-effective and provide patients with peer support.

Factors associated with higher foot self-care competence were female sex, better self-evaluated knowledge about foot self-care, higher value of foot health and sufficient foot health education from health care professionals. These results support those of previous studies among people with DM [22, 23]. DM and RA are long-term health problems that cause foot problems and dysfunction; therefore, the results may be comparable. Women seem to be more interested in their health than men [30], which may explain their higher foot self-care competence levels.

Linear regression analysis confirmed that lower foot self-care competence was reported through four sum variables, the poorer the self-evaluated foot self-care knowledge. This study evaluated basic foot self-care competence, which can be considered a general assessment of foot self-care. In the future, a combination of objective and subjective data collection methods could provide detailed information regarding foot self-care competence deficits. Simulated or observed foot self-care situations could provide evidence of the practical skills required to perform foot self-care. Objective foot self-care knowledge tests can provide information about strengths and deficits. A comprehensive evaluation of foot self-care competence is important for identifying personal development and educational needs to improve foot health.

General population-based information about the benefits of foot health care could support a positive attitude toward foot self-care [31]. In this study, participants with low attitudes toward foot self-care generally considered their foot health as being poorer. Increasing the population-level understanding of effective methods to prevent and promote foot health could help patients with long-term health problems seek methods to alleviate pain or obtain care for foot structural deformities.

Foot self-care is not possible for every patient because of physical restrictions or deficits in foot self-care knowledge [12]. Therefore, in the health care system, there should be equal access to podiatry services when foot self-care is decreased or impossible. The disabling nature of RA damages the joints in the feet as well as the hands and upper extremities. Given the constantly changing nature of RA, individually tailored patient education interventions could promote the management of foot self-care among PwRA. Despite comprehensive patient education, in some cases, self-performed foot care is insufficient and professional podiatric care is needed. Therefore, public positions for podiatrists in health service systems are urgently required to respond to the growing foot health needs of PwRA.

### Strengths and limitations

This study has some limitations. The data were collected from a regional patient association, which limits the generalisability of our results. Participants who were active and interested in their own foot health may have been more likely to respond than those with less interest in their health, which may have created selection bias. To minimise the non-response rate, the data were collected on paper and returned to the researcher in the prepaid envelope. Reminders to respond to the survey were posted on organisations’ websites and on social media. Due to the anonymous nature of the survey, no information about refusal to participate was collected. The item non-response rate was minimal, indicating the usability of the instrument. The psychometric properties of the Competence in Foot Self-care Scale were acceptable. For the category structure, all the response options were used and advanced monotonically. The first component explained 47.7% of the variance in the data, indicating a slightly low level of unidimensionality. However, all items showed acceptable goodness-of-fit values (MnSq) of 0.60–1.36. Person separation was 3.87 (0.94 reliability) and item separation was 6.92 (0.98 reliability), demonstrating wide separation among persons and items.

The evaluation of foot self-care competence was based on self-reported evaluations, which are subjective and prone to bias. The respondents could evaluate their foot self-care competence as higher or lower than that in real life. In addition, apprehension bias, in which participants modify their behaviour or responses due to being observed or the provision of socially desirable responses, could have influenced the results. To control for these biases, each potential participant received detailed information about the study and instructions to respond to the questions on how they subjectively perceive foot self-care competence in their own lives. Despite these limitations, this study provided new perspectives on foot self-care competence in a sample of PwRA.

## Conclusions

In the current study, PwRA evaluated their level of competence in foot self-care as moderate. They considered identification and care of foot problems as important, although they evaluated their skills as limited. A higher self-evaluated level of competence in foot self-care was associated with female sex, higher self-evaluated foot care knowledge, importance of foot health, activity in foot self-care and adequate patient education from health care professionals.

These results indicate the importance of educating PwRA to advance their skills and knowledge of foot self-care. In the future, observational studies focusing on how PwRA perform foot self-care could identify potential gaps in its practical implementation. Furthermore, PwRA could benefit from interventions that increase knowledge of foot self-care together with practical examples or online videos to demonstrate the practice of foot self-care in daily life.

## Data Availability

The datasets used and/or analysed during the current study are available from the corresponding author on reasonable request.

## Abbreviations

DM: Diabetes mellitus
MnSq: Mean square
PwRA: Patients with rheumatoid arthritis
RA: Rheumatoid arthritis
SD: Standard deviation
